# Clinical Assessment of Puressentiel^®^ Muscles and Joints Gel Aromatherapy in the Management of Osteoarthritis-Related Knee Pain

**DOI:** 10.3390/healthcare13020150

**Published:** 2025-01-14

**Authors:** Nicolas Barizien, Perrine Capron, Thomas Mamou, Françoise Louni, Roland Krzentowski, Paul Fogel

**Affiliations:** 1 Service de Réadaptation Fonctionnelle, Hôpital Foch, 92150 Suresnes, France; 2 Clinique du Sport, 134 Bd Saint Marcel, 75005 Paris, France; perrine.capron@gmail.com; 3 Institut Médico-Sportif Préfontaine, 55 Rue Cortambert, 75016 Paris, France; mamou.thomas@gmail.com; 4 Service des Maladies Infectieuses, Hôpital Bichat, 75018 Paris, France; francoise.louni@aphp.fr; 5 Comité Départemental Olympique de Paris, 75013 Paris, France; roland.krzentowski@cdos.fr; 6 ForvisMazars, 94200 Courbevoie, France; paul.fogel@forvismazars.com

**Keywords:** knee osteoarthritis, pain, aromatherapy, topical treatment, Puressentiel^®^ Muscles and Joints gel, WOMAC, essential oils

## Abstract

Introduction: Knee osteoarthritis (OA) is a degenerative joint disease that affects millions globally, causing chronic pain and limited mobility. Pharmacological treatments for OA-related knee pain come with risks, making alternative or complementary therapies attractive. This post-market trial evaluates the efficacy of Puressentiel^®^ Muscles and Joints gel, an aromatherapy gel with 14 essential oils, in managing OA-related knee pain. Method: In this 12-week open-label trial (NCT04736563), participants aged 45–90 with OA-related knee pain applied Puressentiel^®^ Muscles and Joints gel twice daily for 4 weeks, following a 4-week run-in period without treatment. Pain, joint stiffness, and function were assessed using the Western Ontario McMaster Universities Arthritis Index (WOMAC) and Visual Analog Scale (VAS) at baseline, 4 weeks, 8 weeks, and 12 weeks, and oral analgesic intake was recorded daily. Results: Significant improvements in WOMAC and VAS scores were observed during treatment (*p* = 0.0262; *p* < 0.0001, respectively) and sustained 4 weeks post-treatment (*p* = 0.0190; *p* < 0.0001, respectively). Paracetamol intake significantly decreased from baseline to the end of treatment (*p* = 0.0230), though anti-inflammatory intake did not change significantly. No adverse events were reported. Conclusion: Puressentiel^®^ Muscles and Joints gel was well-tolerated, improved WOMAC and VAS scores, and reduced paracetamol use, presenting a viable natural option for pain management in knee OA.

## 1. Introduction

Knee osteoarthritis (OA) is a multifactorial degenerative joint disease that is primarily caused by cartilage degradation due to catabolic factors induced by tissue oxidative stress undergone during aging, obesity, genetic predisposition, and trauma [1,2,3]. Knee OA is estimated to affect 364 million people worldwide, or around 23% of adults over the age of 40, with a higher prevalence in women (29%) than men (16%) [4,5,6].

Progressive cartilage deterioration irritates the subchondral bone and synovium [6,7]. Pain is the first symptom, followed rapidly by a limitation in joint mobility, a source of impaired quality of life (QoL) [8,9,10]. In addition, people who are pain-sensitive have a poorer QoL than those who are not, as do elderly people with osteoarthritis compared to their non-affected counterparts. Reduced QoL can lead to early professional retirement, increased dependence on carers, and social isolation, involving a major economic impact [11].

The guidelines of the European Society for Clinical Aspects of Osteoporosis, Osteoarthritis and Musculoskeletal Diseases (ESCEO) and the Osteoarthritis Research Society International (OARSI) recommend that knee OA be treated using non-pharmacological and pharmacological protocols. Surgery is indicated if these treatments fail [5,12]. Joint stress reduction and weight management are recommended to optimize the efficacy of oral and topical non-steroidal anti-inflammatory drugs (NSAIDs). Oral or intra-articular pharmacological treatments have numerous short- and medium-term side effects. Opioids (oral and transdermal) are strongly discouraged [13]. In contrast to OARSI, the American Academy of Orthopedic Surgeons (AAOS) recommends paracetamol [13,14].

Given these risks, there is growing interest in alternative treatments with fewer side effects, including aromatherapy with essential oils extracted from plants [15,16,17]. As the synovial membrane, the likely source of pain in knee osteoarthritis, is located close to the skin, it is accessible to transcutaneous treatments such as aromatherapy. Essential oils such as peppermint, Roman chamomile, eucalyptus, lavender, ginger, and rosemary have anti-inflammatory and analgesic properties [18]. Numerous preliminary studies have evaluated aromatherapy to reduce pain [15,16,17,19,20,21,22].

The aim of this trial is to evaluate the impact of Puressentiel^®^ Muscles and Joints gel, an aromatherapy gel containing 14 essential oils of natural active ingredients including cajeput, Roman chamomile, clove, eucalyptus globulus, wintergreen, juniper, lavandin grosso, marjoram, peppermint, niaouli, nutmeg, Scots pine, rosemary cineole, and turpentine, on OA-related knee pain.

We hypothesized that twice-daily self-massage with 0.5 g (1 cm^3^) of aromatherapy gel could reduce pain, improve mobility or, alternatively, reduce analgesic consumption, as assessed by VAS and WOMAC scales.

## 2. Materials and Methods

### 2.1. Trial Design and Participants

This was a post-market, interventional, within-subject controlled, open-label, 12-week clinical trial (NCT04736563). Men and women aged 45–90 years with chronic OA knee pain (VAS score of 3 for at least 3 months) with or without pharmacological analgesic therapy were included after they gave their informed consent.

### 2.2. Data Collection

Participants underwent a clinical examination and a standard radiological assessment of the painful knee (performed up to 12 months before inclusion). A Kellgren–Lawrence radiological score of 1–3 was used for the diagnosis of knee OA, and a clinical assessment of mild (grade 1), moderate (grade 2) or severe (grade 3) was made following the EULAR recommendations [23,24]. Participants completed the Algo Functional Western Ontario and McMaster Universities Osteoarthritis Index (WOMAC) questionnaire to measure symptoms and physical disability associated with knee OA and self-assessed pain intensity using the Visual Analog Scale (VAS) of pain at baseline and 4, 8, and 12 weeks. The pain VAS is a unidimensional measure of pain intensity used to record participants’ pain progression or compare pain severity between participants with similar conditions. After a 4-week run-in period during which the participants served as their own control with no investigational product (IP) applied, the participants were allocated the IP Puressentiel^®^ Muscles and Joints gel manufactured by Puressentiel^®^ (lot number F111294H). They were instructed to apply ~0.5 g (1 cm^3^) of Puressentiel^®^ Muscles and Joints gel to the painful knee by massaging the gel into the affected area for 2 to 3 min twice daily (morning and evening) over 4 weeks, after which the IP was no longer applied. A clinical examination was performed at baseline and weeks 4, 8, and 12. At each visit after baseline, participants were asked whether they had experienced adverse events (AEs). Participants completed a daily diary, noting paracetamol, NSAID, and anti-inflammatory intake from baseline to the end of the trial.

### 2.3. Primary and Secondary Endpoints

The primary objective was to evaluate the analgesic effect of Puressentiel^®^ Muscles and Joints gel after dermal application in participants with chronic pain related to knee OA. The primary outcome measure was the evolution of the WOMAC score over time. The Algo Functional WOMAC index evaluates the following three dimensions: pain, joint stiffness, and locomotor function. A 17-point change in total WOMAC score is considered clinically significant (minimum important change [MIC]), with a 10-point change in score considered to be the minimum clinically important difference (MCID) [25,26]. The secondary outcome measures included the evolution in VAS score over time, analgesic intake, and tolerance of Puressentiel^®^ Muscles and Joints gel. The VAS scale ranges from 1 cm to 10 cm, where a score of 1 cm indicates minimal pain and 10 cm is the worst pain. In this trial, a 10% decrease in the VAS score is considered clinically significant. The minimal clinically important improvement (MCII), defined as the smallest change in VAS measurement that signifies an important improvement, is dependent on baseline scores. Tubach et al. 2005 determined that MCIIs for participants with low, intermediate, and high baseline VAS scores were −10.8 mm, −27.4 mm, and −36.6 mm, respectively [27]. Participants recorded daily analgesics and anti-inflammatories. Tolerance of Puressentiel^®^ Muscles and Joints gel was assessed in terms of AEs reported by the participants.

### 2.4. Statistical Analyses

Continuous variables were described by their mean and standard deviation (SD), and compared using Student’s *t*-test. Categorical variables were described by number and percentage, and compared using the Chi2 test. All tests were bilateral, with a *p*-value of <0.05 considered statistically significant. A mixed model was used to study the evolution of WOMAC, VAS, and intake of analgesics. The mean values at 4 and 8 weeks were compared using Student’s *t*-test. The JMP statistical software, version 17.0 (Cary, NC, USA: SAS Institute Inc.), was used to perform all analyses.

## 3. Results

### 3.1. Participant Demographics

There were 30 participants included in this trial. The mean age was 55.3 ± 2.2 years, and 53% were female. The mean body mass index (BMI) was normal at 23.5 ± 8 kg/m^2^ (Table 1). The mean clinical class was 1.33 ± 0.11, with a Kellgren–Lawrence radiological score of 1.93 ± 0.16. Participants had experienced knee pain for an average of 4.3 ± 0.7 years.

### 3.2. Primary Endpoint

**Figure 1:**
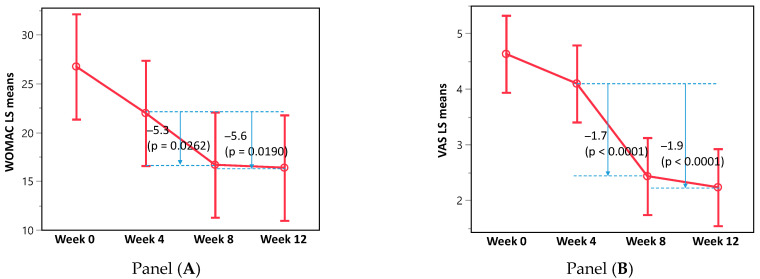
WOMAC least squares (LS) mean score (**left panel A**) and VAS scores (**right panel B**) decreased significantly during Puressentiel^®^ Muscles and Joints gel treatment (weeks 4–8) and remained significantly decreased post-treatment (weeks 4–12).

The average WOMAC score decreased significantly after 4 weeks of treatment with Puressentiel^®^ Muscles and Joints gel between weeks 4 and 8 (*p* = 0.0262) and was maintained for a further 4 weeks post-treatment (between weeks 4 and 12; *p* = 0.0190) (Figure 1A). However, the maximum decrease in score of −5.6 points (MCID of 5.6) between weeks 4 and 12 was not considered clinically significant as it was lower than the 17-point change established as the MIC cut off, and the 10 points required for the MCID cut off [25,26].

### 3.3. Secondary Endpoints

Similarly, the VAS score decreased significantly during treatment between weeks 4 and 8 (*p* = 0.0001) and remained significantly decreased post-treatment (between weeks 4 and 12; *p* = 0.0001) (Figure 1B). As the −1.9 cm decrease in VAS score represented a >10% change in VAS score, it was considered clinically significant. Furthermore, a 19 mm decrease in the VAS score exceeds the MCII of −10.8 mm for OA-related knee pain in individuals with a baseline VAS score of 30–51 mm [27].

**Figure 2:**
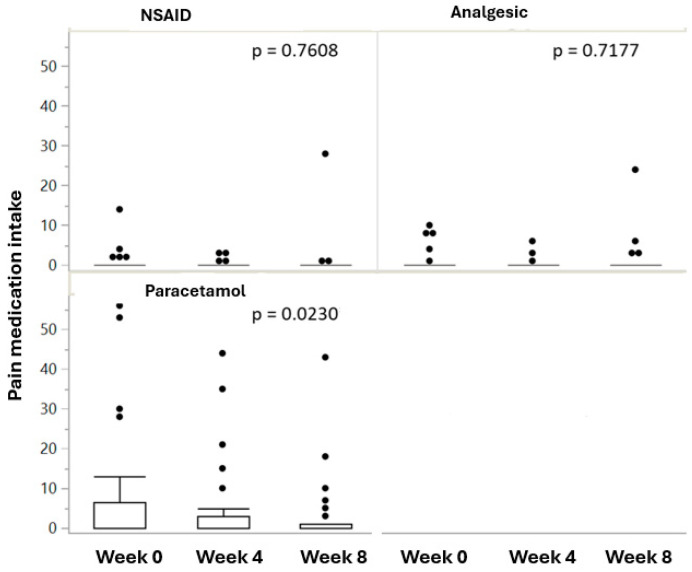
Paracetamol intake decreased significantly between baseline and week 8 (*p* = 0.0230), but not weeks 4–8, nor between baseline and week 4. The decrease in NSAID and analgesic intake was not significant.

Over a third of participants (37%) were taking paracetamol at baseline, with fewer participants taking NSAIDs (16%) and analgesics (16%). During run-in, the proportion of participants taking paracetamol, analgesics, and NSAIDs decreased (7%, 7%, and 4%, respectively). By the end of week 8, after 4 weeks of Puressentiel^®^ Muscles and Joints gel treatment, the intake of paracetamol and NSAIDs further decreased by 7% and 3%, respectively, while the intake of analgesics increased by 3%. However, the decrease in the proportion of participants taking pain medication was not statistically significant due to the small sample size. The number of intakes of paracetamol per participant decreased significantly between week 0 and week 8 (*p* = 0.0230), but not during treatment (between week 4 and week 8), nor during run-in (between week 0 and week 4 (Figure 2). There was no significant decrease in the number of intakes of analgesics or NSAIDs.

**Figure 3:**
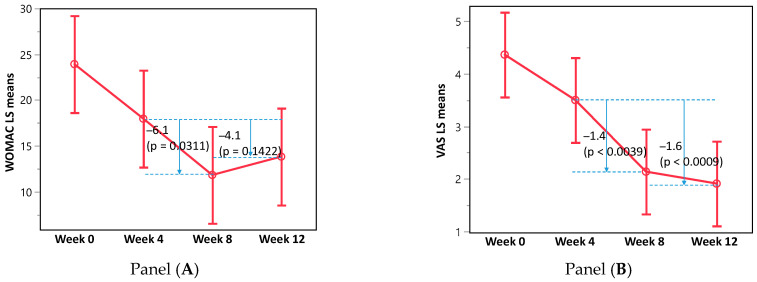
WOMAC LS mean score (**left panel A**) and VAS scores (**right panel B**) decreased significantly during Puressentiel^®^ Muscles and Joints gel use after the exclusion of participants with high intake of oral pain medication (weeks 4–8). Unlike the VAS score, the WOMAC score decrease was not significant in weeks 4–12.

When the WOMAC and VAS scores were recalculated after excluding eight participants who had ≥10 oral pain medication intakes, the decrease in the WOMAC and the VAS remained significant during treatment (between weeks 4–8 weeks; WOMAC, *p* = 0.0311; VAS, *p* = 0.0039) (Figure 3). However, the decrease in the WOMAC score was not significant in weeks 4–12 (*p* = 0.1411), while the decrease in the VAS score remained significant (*p* = 0.0009).

None of the participants reported an AE during the trial.

## 4. Discussion

In this trial, the application of Puressentiel^®^ Muscles and Joints gel twice daily significantly improved the WOMAC and VAS scores over time. The participants’ paracetamol intake was decreased, but not the anti-inflammatory intake. When participants with a high intake of pain medication were excluded from the analyses, the WOMAC and VAS scores were still significantly improved, with the improvement in the VAS score sustained after Puressentiel^®^ Muscles and Joints gel was no longer applied. While the improvement in the WOMAC scores did not reach clinical significance (MCID of 5.6), the change in the VAS scores was considered a clinically important improvement. There were no AEs reported in this trial.

The results from this trial support the findings from other studies using aromatherapy to manage OA-related knee pain in which significant improvements in WOMAC and VAS scores were also reported [17,19,21,28]. In a controlled trial, Efe Arslan et al. 2018 compared the WOMAC and VAS scores of individuals with knee OA who had an aromatherapy massage, a conventional massage with olive oil, or standard of care treatment over 3 weeks. The aromatherapy treatment consisted of sweet almond, apricot kernel, lavender, eucalyptus, and ginger oil, and was administered by a healthcare professional (HCP) during a 15-min massage [17]. After 1 week, the WOMAC index and VAS scores were significantly improved for the aromatherapy massage compared with the conventional massage and control, and remained so over a 3-week duration. Pehlivan et al. 2019 also investigated the effects of aromatherapy massage on the pain, functional state, and QoL of elderly individuals with knee OA. They demonstrated that twice-weekly 20-min aromatherapy massages over 3 weeks with black seed, ginger, and rosemary oils, administered by an HCP, significantly improved the WOMAC and VAS scores and QoL [21]. Furthermore, in a double-blind, randomized, placebo-controlled trial, Yip et al. 2008 assessed the impact of six 30-min HCP-administered aromatherapy massage sessions, with ginger and sweet orange oil in the elderly with moderate-to-severe knee pain over 3 weeks. In 1 week, the WOMAC pain and physical function scores in the aromatherapy massage group significantly improved compared to the conventional massage, and to the control group. However, the effect was no longer significant after 3 weeks, and no difference between the groups regarding QoL was observed [28].

As observed in other published trials, the results from our clinical trial support the use of aromatherapy as a natural pain management treatment option for individuals with knee OA. While the specific mechanism of action of Puressentiel^®^ Muscles and Joints gel for reducing pain has not been established, the analgesic properties of chamomile, clove, eucalyptus, and peppermint, all of which are components of the gel, have been reported [17,29]. The participants in our trial had an average Kellgren–Lawrence radiological grade of 1.93 ± 0.16. Grade 2 indicates mild knee OA and is associated with occasional pain and stiffness, particularly at the end of the day. This was reflected by the average WOMAC index and VAS scores at week 4 (22 and 4, respectively). However, it should be noted that during run-in without Puressentiel^®^ Muscles and Joints gel, the WOMAC and VAS scores also decreased. This decrease may be partly due to the participants complying with the request to walk regularly (structured land exercise program), leading to improved WOMAC and VAS scores. While the essential oils used in the aromatherapy treatment in other trials were not identical to those of Puressentiel^®^ Muscles and Joints gel, many of the essential oils used in the trials were reported to have anti-inflammatory or analgesic properties [18]. A notable difference between our trial and other reported aromatherapy trials is that the aromatherapy massage was self-administered for ~3 min, without the intervention of an HCP. The participants in some of the other trials may have experienced more OA-related knee pain, as evidenced by higher baseline WOMAC and VAS scores [17,21].

Chronic pain associated with knee OA requires treatment as it impacts QoL, potentially leading to debilitating consequences. Such consequences can include discomfort that limits daily activities, reduced social interactions, dependence on caregivers, and for some, a decline in mental health. While pharmacological treatment is a recommended option for pain management, the risks are numerous and well-documented, including stroke, GI ulcers, and renal and liver damage, making long-term use inadvisable. Before taking recourse in pharmacological treatment, and as recommended by ESCEO, it is important that individuals with knee OA are educated about the disease, follow a structured exercise program, reduce joint stress, and manage their weight. Nevertheless, knee OA can also affect those with a normal BMI, such as the participants of this trial with a BMI of 23. For individuals with OA-related knee pain, Puressentiel^®^ Muscles and Joints gel could be a natural alternative or complement to pharmacological pain management, especially if pharmacological therapy is not recommended for them either because of comorbidities or contraindicated concomitant medications. Interestingly, after 4 weeks of treatment with Puressentiel^®^ Muscles and Joints, gel pain (the VAS score) was reduced by 45% (measured at week 8), which was similar to the findings for the NSAID topical diclofenac gel, where chronic knee pain was decreased by at least 50% after 6 weeks of treatment [30].

### Strengths and Limitations

One of the strengths of this trial is that it was within-subject-controlled. However, it was not randomized, and the sample size was insufficient to statistically demonstrate that fewer participants used pain medication during treatment with Puressentiel^®^ Muscles and Joints gel. Additionally, the potential bias of massage for pain relief and a placebo effect cannot be discounted. Skin type (oily/dry) was not taken into consideration and may also impact efficacity. Furthermore, the differences in the levels of physical activity between the participants during the trial, and in the analgesics taken, may also be a source of bias.

## 5. Conclusions

WOMAC and VAS scores significantly improved when Puressentiel^®^ Muscles and Joints gel was used to manage the pain symptoms of knee OA. However, while the improvement in the VAS scores was clinically important, the change in the WOMAC scores was not clinically relevant. Paracetamol use significantly decreased, but not that of anti-inflammatories. Puressentiel^®^ Muscles and Joints gel was well-tolerated. Puressentiel^®^ Muscles and Joints gel may be used as a natural pain management option for individuals with mild-to-moderate knee OA, allowing them to avoid the risks associated with long-term use of pharmacological treatment. Puressentiel^®^ Muscles and Joints gel may be of particular interest to individuals for whom pharmacological therapy is not recommended because of comorbidities or concomitant medications.

## Figures and Tables

**Table 1:** Summary of participant characteristics at baseline.

Characteristic	Value
Age—mean ± SD, years	55.3 ± 2.2
Sex—n (%)	
Female	16 (53%)
Male	14 (47%)
Weight—mean ± SD, Kg	66.9 ± 2.7
Height—mean ± SD, m	1.68 ± 0.02
BMI—mean ± SD, years Kg/m^2^	23.5 ± 8
Duration of knee pain—mean ± SD, years	4.3 ± 0.7
Kellgren–Lawrence radiological score—mean ± SD	1.93 ± 0.16
Clinical class– mean ± SD	1.33 ± 0.11

BMI = body mass index; SD = standard deviation.

## Data Availability

The data are contained within the article.
